# The effect of postural orientation on body composition and total body water estimates produced by smartwatch bioelectrical impedance analysis: an intra- and inter-device evaluation

**DOI:** 10.2478/joeb-2024-0010

**Published:** 2024-08-05

**Authors:** Anabelle Vallecillo-Bustos, Abby T. Compton, Sydney H. Swafford, Megan E. Renna, Tanner Thorsen, Jon Stavres, Austin J. Graybeal

**Affiliations:** 1 School of Kinesiology and Nutrition, University of Southern Mississippi, Hattiesburg, MS, USA; 2 School of Psychology, University of Southern Mississippi, Hattiesburg, MS, USA

**Keywords:** Wearables, smartwatch, digital health, sensors, body composition, body water, digital anthropometrics

## Abstract

Advances in wearable technologies now allow modern smartwatches to collect body composition estimates through bioelectrical impedance techniques embedded within their design. However, this technique is susceptible to increased measurement error when postural changes alter body fluid distribution. The purpose of this study was to evaluate the effects of postural orientation on body composition and total body water (TBW) estimates produced by smartwatch bioelectrical impedance analysis (SWBIA) and determine its agreement with criterion measures. For this cross-sectional evaluation, 117 (age: 21.4±3.0 y; BMI: 25.3±5.7 kg/m^2^) participants (F:69, M:48) completed SWBIA measurements while in the seated, standing, and supine positions, then underwent criterion dual-energy X-ray absorptiometry (DXA) and bioelectrical impedance spectroscopy (BIS) assessments. In the combined sample and females, body fat percent, fat mass, and fat-free mass using SWBIA were significantly different between the supine and standing positions (all p<0.001), though group level agreement with DXA was similar across positions. Supine SWBIA TBW estimates were significantly different between seated and standing estimates (all p≤0.026), but further analyses revealed that this was driven by the supine and seated differences observed in females (p=0.003). SWBIA TBW demonstrated similar group and individual level agreement with BIS across body positions with slight improvements observed during seated and supine assessments for females and males, respectively. SWBIA may demonstrate slight intra- and inter-device differences in body composition and TBW when measured across postural orientations, though further evaluations in external/clinical samples are necessary. While sex/position-specific guidelines may improve precision, these findings highlight the importance of standardized body positioning when using SWBIA.

## Introduction

Continual advances in wearable technology now allow most modern wearables to monitor complex health information [1] without increasing cost or size, and without sacrificing complexity or portability [2]. Moreover, the desire for wearable health information is shared by both clinicians and consumers alike, evident by recent reports showing that more than 50% of US adults own a smartwatch and would be willing to share their digital health information with their healthcare providers [2]. As such, wearable technologies continue to revolutionize both the health and fitness industries by overcoming the economic and geographical limitations inherent to standard health assessments.

Given that body composition estimates are associated with health status and are highly desired by consumers, it is unsurprising that recent developments have equipped smartwatches to measure components of body composition. Specifically, smartwatches have adapted standard bioelectrical impedance analysis (BIA) to produce real-time estimates of body composition using electrodes embedded within the smartwatch’s hardware. While the ability to monitor such variables without a technician present allows consumers greater autonomy over their health [3], evidence supporting the accuracy of smartwatch bioelectrical impedance (SWBIA) remains mixed. For instance, SWBIA has been unable to demonstrate equivalence (using equivalence and paired null-hypothesis significance tests), nor precision (ex: root mean square error [RMSE]), when compared to more sophisticated methodologies such as dual-energy X-ray absorptiometry (DXA) and 4-compartment models; however, performance has been shown to improve following systematic correction [1,3]. While the concerns necessitating these corrections are thought to be attributed to the exclusion of body regions that typically demonstrate greater fat mass (FM) (i.e., trunk and lower body), it is possible that other systematic complications contribute to, or compound upon, these methodological disagreements.

Specifically, BIA calculates body composition by injecting alternating currents into the body that indirectly assess fluid distribution [4]. As such, BIA-derived body composition estimates are contingent upon the user’s postural orientation and corresponding fluid redistributions [5]. For example, fluid is concentrated in the inferior portion of the body during standing whereas in the supine position, fluids are more evenly distributed — altering the flow of the injected currents and resultant body composition estimates [6]. Thus, to avoid undue error, it is imperative that newly developed BIA methods are developed and evaluated against criterion methods that employ similar body positions. However, BIA-derived estimates are most often evaluated against supine-specific criterion methods (a standard position in clinical settings) despite the fact the majority of BIA methods are performed while standing. Moreover, sex-specific body composition distribution patterns may have compounding effects on position-specific SWBIA measurement error. Specifically, greater FM accumulation occurs in the trunk and lower body of males and females, respectively, whereas fat-free mass (FFM) is greater in the male upper body (where the SWBIA currents are injected) when compared to females. Given the overlap between body composition and fluid distribution patterns, it is likely that these sex-specific tissue arrangements are implicated in the postural fluid-shifts that increase the measurement error of BIA.

Unfortunately, most clinically accessible BIA devices are unable to address the aforementioned issues due to their rigid designs. However, SWBIA is in a unique position to improve upon these concerns given that it can be adapted to collect body composition estimates from virtually any postural orientation. Furthermore, it is critical for users to follow a standardized protocol during self-assessment, yet most devices do not give explicit recommendations about body positioning; leaving this up to the consumer’s discretion at each measurement period. As such, determining the optimal SWBIA measurement position would allow for more informed measurement guidelines that account for the effects of fluid distribution; particularly if distributions differ by sex. Therefore, the purpose of this study was to investigate the effects of postural orientation on SWBIA-derived body composition estimates and their position-specific agreement with DXA. A secondary aim of this investigation was to evaluate the effects of body position on the agreement between SWBIA and bioelectrical impedance spectroscopy- (BIS) derived estimates of total body water (TBW).

## Materials and methods

### Participants

A total of 126 participants were prospectively recruited for this cross-sectional evaluation. Participants were excluded if they had a significant amount of internal metal such as a metal plate or a complete joint replacement (to avoid interference with the injected currents from bioelectrical devices and artifact using DXA); had a pacemaker or any other electrical implant; were missing any limbs or part of a limb; or were pregnant; trying to become pregnant; or breast-feeding or lactating. Nine participants were excluded due to scheduling conflicts and thus, a diverse sample of 117 (42 Non-Hispanic White; 37 Non-Hispanic Black; 32 Asian; 6 Hispanic White) males and females (48 M; 69 F) between the ages of 18 and 39 (age: 21.4 ± 3.0 y; BMI: 25.3 ± 5.7 kg/m^2^) completed this study in its entirety.

### Procedures

Participants reported to the laboratory following an ≥ 8 h overnight fast from food, beverage, supplements and medication and after abstaining from exercise for ≥ 24 h. Upon arrival, participants were instructed to remove all external metal and accessories (jewelry, shoes, watches, etc.), and any excessive clothing, so that only tight fitting athletic clothing was worn during testing. Then, participants underwent measurements of height using a stadiometer, body mass using a calibrated digital scale (SECA 769, Hamburg, Germany), and body composition assessments using DXA (Lunar iDXA, General Electric, Boston, MA, USA), BIS (SFB7, Impedimed^®^, Carlsbad, CA, USA), and SWBIA (Galaxy Watch 4^®^, Samsung^®^, Suwon, South Korea). To ensure the most equitable comparisons, the body mass collected from the calibrated digital scale was the body mass used for both SWBIA and DXA. As such, FM was calculated as: body mass x (BF%/100); and FFM was calculated as body mass minus FM.

### Bioelectrical impedance analysis and dual-energy X-ray absorptiometry

The methods used for SWBIA [3], BIS [4], and DXA [7] have been described in detail elsewhere, and a summary of these procedures are presented in the following paragraphs with additional information unique to this study provided in greater detail. Moreover, this study is a secondary analysis that extends from a larger line of investigation (ClinicalTrials.gov; NCT05885672); though the dependent variables presented in this study are unique to the current investigation.

### Smartwatch bioelectrical impedance analysis

Body composition estimates from a single-frequency, hand- to-hand, tetrapolar SWBIA were produced using a common consumer grade 40 mm smartwatch paired to a mobile application (Samsung Health^®^; Samsung^®^, Suwon, South Korea). Importantly, this device has previously demonstrated acceptable test-retest reliability [3]. Using four individual electrodes positioned underneath the watch face (two electrodes in contact with the user’s left wrist) and within watch frame (two electrodes in contact with the user’s right middle and ring finger) this SWBIA injects alternating currents to measure the impedance of the user’s upper body FFM at a frequency of 50 kHz and subsequently generates estimates of whole-body BF%, FM, and TBW from proprietary algorithms embedded within the watch’s software [1,3]. Importantly, this SWBIA is equipped with an internal analog front-end chip and has signal electrodes positioned inside the reference electrodes to account for the greater impedance and current concentrations inherent to its small and narrow electrode configuration [1,3].

Prior to testing, each participants’ age, sex, height, and weight were entered into the affiliated mobile application which was subsequently synced with the SWBIA. Then, the smartwatch was fastened on the participant’s left wrist so that the bottom electrodes were in complete contact with the skin. After the watch was successfully secured, a series of three separate measurements were performed while participants were 1) seated, 2) standing, or 3) supine. For the seated position, participants were instructed to sit up straight in a standard chair with feet flat on the ground so that their legs were approximately 90°. For the supine position, participants were positioned on an exam table with their backs flat and hands to their side until the measurement required movement of the hands. For the standing position, participants were simply asked to stand upright. Participants remained in each position for ≥ 10 min prior to testing so that body fluid could be adequately redistributed. The smartwatch was removed and repositioned prior to each measurement.

After remaining in each position for ≥ 10 min, participants were instructed to pronate their left wrist, supinate their right wrist, and laterally raise both arms. Participants were then instructed to bring to their hands together as to make a “circle” or “hoop” with their arms, and to position the tips of their right middle and ring fingers so that they were in firm contact with the electrodes extending from the watch’s frame. Participants were also instructed to bend their left wrist so that contact between the right fingers and the surface of the left hand could be avoided. For the seated and standing conditions, participants raised their arms until parallel to the floor. For the supine position, participants raised their arms upward to a comfortable point where they could see the watch’s interface — representing the position most likely to be performed in practice. After the participant’s fingers were placed against the electrodes, participants remained still for 15 seconds while the measurement was completed.

### Bioelectrical impedance spectroscopy

TBW estimates were collected using a validated [8] multi-frequency, hand-to-hand, tetrapolar BIS (SFB7, Impedimed^®^, Carlsbad, CA, USA). Prior to testing, participants were instructed to lay supine for ≥ 10 min while two adhesive electrodes were placed on their left posterior wrist and hand and left anterior ankle and foot. The hand and foot electrodes were placed approximately 5 cm distal to their limb-specific proximal electrodes [9] in accordance with the manufacturer guidelines. Then, a single measurement was performed to produce estimates of TBW at a frequency of 50 kHz; which was selected 1) to match the frequency of the SWBIA, allowing for the most equitable comparisons between SWBIA and BIS; and 2) because 50 kHz represents the standard frequency used in research and practice for both single- and multi-frequency bioelectrical impedance devices [4,10]. The sex, body density, body proportion, and hydration coefficients were the default recommendations established by the manufacturer as used in prior investigations [4,10]. BIS was calibrated daily prior to testing, and visual inspection of Cole plots were performed following each measurement. For reasons unbeknownst to the investigators nor the manufacturer, the BIS device used in this study experienced technical difficulties with the device interface that required the device to be serviced by the manufacturer. While this did not have an impact on any of the data reported in the present study, BIS was unavailable for testing during the visits of 10 individual participants, and a single participant had Cole plots outside of the acceptable bounds (unrelated to the technical difficulties). Thus, TBW data was missing/omitted for 11 participants. As such, 106 participants (62 F, 44 M) were used to assess the agreement between the SWBIA and BIS-derived TBW as opposed to the 117 used to evaluate the agreement between SWBIA and DXA-derived body composition estimates.

### Dual-energy X-ray absorptiometry

A Lunar iDXA with version 18 enCORE software was used to produce estimates of whole-body BF%, FM, and FFM. Participants were positioned on the DXA according to manufacturer’s recommended guidelines [7] and reflection scanning procedures were performed for larger participants [11].

### Statistical analyses

Using a small-to-medium effect size of d = 0.30 for a two-tailed paired null hypothesis significance test, it was determined that 90 participants would yield 80.4% power at an α = 0.050. Differences in the descriptive characteristics by sex were assessed using independent samples t-tests. Repeated measures ANOVA with Bonferroni-Holm corrections for multiple comparisons were used to determine the differences in the SWBIA-derived body composition estimates by body position. The mean difference (MD) between SWBIA and the criterion DXA and BIS were calculated as the SWBIA estimate in question minus the estimates produced by either DXA (BF%, FM, FFM) or BIS (TBW) and evaluated using null hypothesis significance and equivalence tests. The equivalence regions used for equivalence testing were defined as ±1.0% for BF%; ±1.0 kg for FM and FFM; and ±1.0 L for TBW. Additionally, the agreement between SWBIA and the criterion DXA and BIS were evaluated using coefficients of determination (R2), Deming regression, Bland-Altman analyses, RMSE, and concordance correlation coefficients (CCC). Agreement between the predicted values and the line of identity produced using weighted Deming regression was determined if 95% confidence intervals (95%CI) for the intercept and slope contained the values 0 and 1, respectively. The 95% limits of agreement (LOA) and proportional biases were determined using Bland-Altman and linear regression techniques. All evaluations were conducted for the combined sample and by sex. Data were normally distributed based on the results of Shapiro-Wilk and visual inspection of Q-Q plots. Statistical significance was accepted at p < 0.050 and data were analyzed using R version 4.1.2 and Microsoft Excel version 16.

### Informed consent

Informed consent has been obtained from all individuals included in this study.

### Ethical approval

The research related to human use has been complied with all relevant national regulations, institutional policies and in accordance with the tenets of the Helsinki Declaration, and has been approved by the authors’ institutional review board or equivalent committee.

## Results

### Participants

Participant characteristics are presented in **Table 1**. Of the 117 participants, 66 were classified as normal weight (56.4%) and 51 were classified as having either overweight (n = 33; 28.2%) or obesity (n = 18; 15.4%). Additionally, 35.9% were White, 31.6% were Black, 27.4% were Asian, and 5.1% were Hispanic. All descriptive characteristics were significantly different between males and females (all p ≤ 0.008) with the exception of age (p = 0.391) and body mass index (BMI; p = 0.590).

**Table 1.** Participant characteristics.

	Total (n = 117)	Female (n = 69)	Male (n = 48)
	**Mean ± SD**	**Mean ± SD**	**Mean ± SD**
Age (y)	21.4 ± 3.0	21.2 ± 1.8	21.7 ± 4.1
Height (cm)	168.2 ± 8.9	163.4 ± 5.9*	175.2 ± 7.8
Weight (kg)	71.9 ± 18.1	68.2 ± 17.6*	77.1 ± 17.7
BMI (kg/m^2^)	25.3 ± 5.7	25.5 ± 6.4	25.0 ± 4.5
Body fat (%)	28.6 ± 10.3	33.6 ± 8.9*	21.5 ± 7.6
Fat mass (kg)	21.2 ± 12.3	24.1 ± 13.2*	17.2 ± 9.6
FFM (kg)	50.7 ± 12.0	44.2 ± 6.7*	60.0 ± 11.8
TBW (L)	37.0 ± 8.1	33.4 ± 6.0*	42.1 ± 7.9
	**N (%)**	**N (%)**	**N (%)**
**Race**			
White	42 (35.9%)	29 (42.0%)	13 (27.1%)
Black	37 (31.6%)	25 (36.2%)	12 (25.0%)
Asian	32 (27.4%)	10 (14.5%)	22 (45.8%)
Hispanic	6 (5.1%)	5 (7.2%)	1 (2.1%)
**Weight Status**			
Normal	66 (56.4%)	40 (58.0%)	26 (54.2%)
Overweight	33 (28.2%)	18 (26.1%)	15 (31.3%)
Obese	18 (15.4%)	11 (15.9%)	7 (14.6%)

1 Data are presented as mean ± standard deviation or as n (percent of the column total). SD: standard deviation; BMI: body mass index; FFM: fat-free mass; TBW: total body water.

* significantly difference from males at p < 0.050.

### Effects of body position on body composition and total body water estimates produced by SWBIA

The means for SWBIA body composition and TBW estimates by body position are presented in **Table 2** and **Table 3**, respectively. BF% (F = 5.67; p = 0.004), FM (F = 6.35; p = 0.002), FFM (F = 6.35; p = 0.002), and TBW (F = 3.99; p = 0.020) were significantly different by body position in the combined sample. Post hoc analyses showed that all body composition estimates, with the exception of TBW (p = 0.051), were all significantly different between standing and supine positions (all p < 0.001). However, simple effects tests revealed data consistent with differences between standing and supine TBW (MD: 0.13 L, MD^95%CI^: 0.02, 0.25; d: 0.21, d^95%CI^: 0.03, 0.39; p = 0.026). TBW estimates were significantly different between the seated and supine positions (p = 0.015). There were no significant differences between the sitting and standing positions (all p ≥ 0.195).

**Table 2.** Agreement between SWBIA and DXA-derived body composition estimates by body position.

	Mean (95%CI)	MD (95%CI)	R^2^	RMSE	CCC
**Combined (N = 117) BF%**					
DXA	28.6 (26.7, 30.5)				
SWBIA_Seated_	29.0 (27.4, 30.7)	.43 (-.38, 1.23)	.82	4.40	.90
SWBIA_Stand_	28.9 (27.2, 30.6)	.30 (-.50, 1.10)	.82	4.37	.90
SWBIA_Supine_	29.2 (27.5, 30.9)*	.62 (-.17, 1.42)	.82	4.37	.90
**FM (kg)**					
DXA	21.2 (19.0, 23.5)				
SWBIA_Seated_	21.6 (19.5, 23.6)	.31 (-.28, .89)	.94	3.18	.96
SWBIA_Stand_	21.5 (19.4, 23.5)	.22 (-.37, .80)	.93	3.19	.96
SWBIA_Supine_	21.7 (19.6, 23.8)*	.45 (-.12, 1.03)	.94	3.15	.96
**FFM (kg)**					
DXA	50.7 (48.5, 52.8)				
SWBIA_Seated_	50.3 (48.3, 52.4)	-.31 (-.89, .28)	.93	3.18	.96
SWBIA_Stand_	50.4 (48.4, 52.5)	-.22 (-.80, .37)	.93	3.19	.96
SWBIA_Supine_	50.2 (48.1, 52.3)*	-.45 (-1.03, .12)	.93	3.15	.96
**Females (n = 69) BF%**					
DXA	33.6 (31.4, 35.7)				
SWBIA_Seated_	34.2 (32.4, 35.9)	.56 (-.35, 1.47)	.82	3.81	.89
SWBIA_Stand_	33.9 (32.2, 35.7)	.34 (-.60, 1.27)	.82	3.88	.89
SWBIA_Supine_	34.4 (32.7, 36.1)*	.81 (-.11, 1.73)	.83	3.89	.88
**FM (kg)**					
DXA	24.1 (20.9, 27.2)				
SWBIA_Seated_	24.3 (21.4, 27.3)	.26 (-.35, .87)	.97	2.54	.98
SWBIA_Stand_	24.2 (21.3, 27.1)	.12 (-.50, .74)	.97	2.57	.98
SWBIA_Supine_	24.5 (21.6, 27.5)*	.44 (-.17, 1.05)	.97	2.55	.98
**FFM (kg)**					
DXA	44.2 (42.6, 45.8)				
SWBIA_Seated_	43.9 (42.3, 45.5)	-.26 (-.87, .35)	.86	2.54	.93
SWBIA_Stand_	44.0 (42.5, 45.6)	-.12 (-.74, .50)	.86	2.57	.93
SWBIA_Supine_	43.7 (42.1, 45.3)*	-.44 (-1.05, .17)	.86	2.55	.93
**Males (n = 48) BF%**					
DXA	21.5 (19.3, 23.7)				
SWBIA_Seated_	21.7 (19.9, 23.5)	.24 (-1.26, 1.74)	.56	5.13	.72
SWBIA_Stand_	21.7 (19.9, 23.5)	.25 (-1.22, 1.72)	.56	5.00	.73
SWBIA_Supine_	21.8 (20.0, 23.7)	.35 (-1.11, 1.81)	.57	4.98	.74
**FM (kg)**					
DXA	17.2 (14.4, 20.0)				
SWBIA_Seated_	17.5 (15.0, 20.1)	.37 (-.77, 1.52)	.83	3.93	.91
SWBIA_Stand_	17.5 (15.0, 20.0)	.36 (-.79, 1.50)	.83	3.90	.91
SWBIA_Supine_	17.6 (15.1, 20.2)	.47 (-.65, 1.59)	.84	3.85	.91
**FFM (kg)**					
DXA	60.0 (56.5, 63.4)				
SWBIA_Seated_	59.6 (56.6, 62.6)	-.37 (-1.52, .77)	.90	3.93	.94
SWBIA_Stand_	59.6 (56.6, 62.6)	-.36 (-1.50, .79)	.89	3.90	.94
SWBIA_Supine_	59.5 (56.5, 62.5)	-.47 (-1.59, .65)	.90	3.85	.94

1 Means and (95% confidence intervals) are presented for each body composition estimate. 95%CI: 95% confidence interval; MD: mean difference; R2: coefficient of determination; RMSE: root mean square error; CCC: concordance correlation coefficients; BF%: body fat percentage; DXA: dual-energy X-ray absorptiometry; SWBIA: smartwatch bioelectrical impedance analysis; FM: fat mass; FFM: fat-free mass.

* significantly different from the standing position at p<0.050

**Table 3.** Agreement between SWBIA and BIS-derived estimates of total body water by body position.

	Mean (95%CI)	MD (95%CI)	R^2^	RMSE	CCC
**Combined (N = 106)**					
BIS	37.0 (35.4, 38.5)				
SWBIA_Seated_	37.2 (35.7, 38.7)	.08 (-.46, .62)	.88	2.80	.94
SWBIA_Stand_	37.2 (35.6, 38.7)	.05 (-.49, .59)	.89	2.79	.94
SWBIA_Supine_	37.1 (35.5, 38.6)*,^†^	-.05 (-.59, .49)	.89	2.79	.94
**Female (N = 62)**					
BIS	33.4 (31.9, 34.9)				
SWBIA_Seated_	32.6 (31.3, 33.8)	-.78 (-1.43, -.14)^‡^	.82	2.64	.89
SWBIA_Stand_	32.5 (31.3, 33.8)	-.85 (-1.49, -.20)^‡^	.82	2.66	.89
SWBIA_Supine_	32.4 (31.1, 33.7)^†^	-.96 (-1.61, -.31)^‡^	.81	2.72	.88
**Male (N = 44)**					
BIS	42.1 (39.6, 44.5)				
SWBIA_Seated_	43.9 (41.7, 46.1)	1.29 (.46, 2.13)^‡^	.88	3.02	.92
SWBIA_Stand_	43.9 (41.7, 46.0)	1.32 (.50, 2.13)^‡^	.89	2.96	.93
SWBIA_Supine_	43.8 (41.6, 46.0)	1.23 (.43, 2.04)^‡^	.89	2.88	.93

1 Means and (95% confidence intervals) are presented for each body composition estimate. 95%CI: 95% confidence interval; MD: mean difference; R^2^: coefficient of determination; RMSE: root mean square error; CCC: concordance correlation coefficients; BF%: body fat percentage; DXA: dual-energy X-ray absorptiometry; SWBIA: smartwatch bioelectrical impedance analysis.

* significantly different from the standing position at p<0.050;

† significantly different from the seated position at p<0.050;

‡ significant mean difference from BIS at p<0.050

For females, BF% (F: 6.79; p = 0.002), FM (F: 7.10; p = 0.001), and FFM (F: 7.11; p = 0.001) were significantly different across body positions, with post hoc tests revealing differences between standing and supine estimates (all p < 0.001). Despite a non-significant omnibus model for female TBW (F = 2.80; p = 0.064), simple effects tests revealed data consistent with differences between seated and supine TBW estimates (MD: 0.17 L, MD^95%CI^: 0.06, 0.28; d: 0.38, d^95%CI^: 0.13, 0.62; p = 0.003). There were no significant differences between the seated and standing positions for females (all p ≥ 0.153). No significant differences were observed across positions in the male only sample (all p ≥ 0.290).

### Agreement between SWBIA and DXA-derived body composition estimates by body position

**Fig 1a-d.**
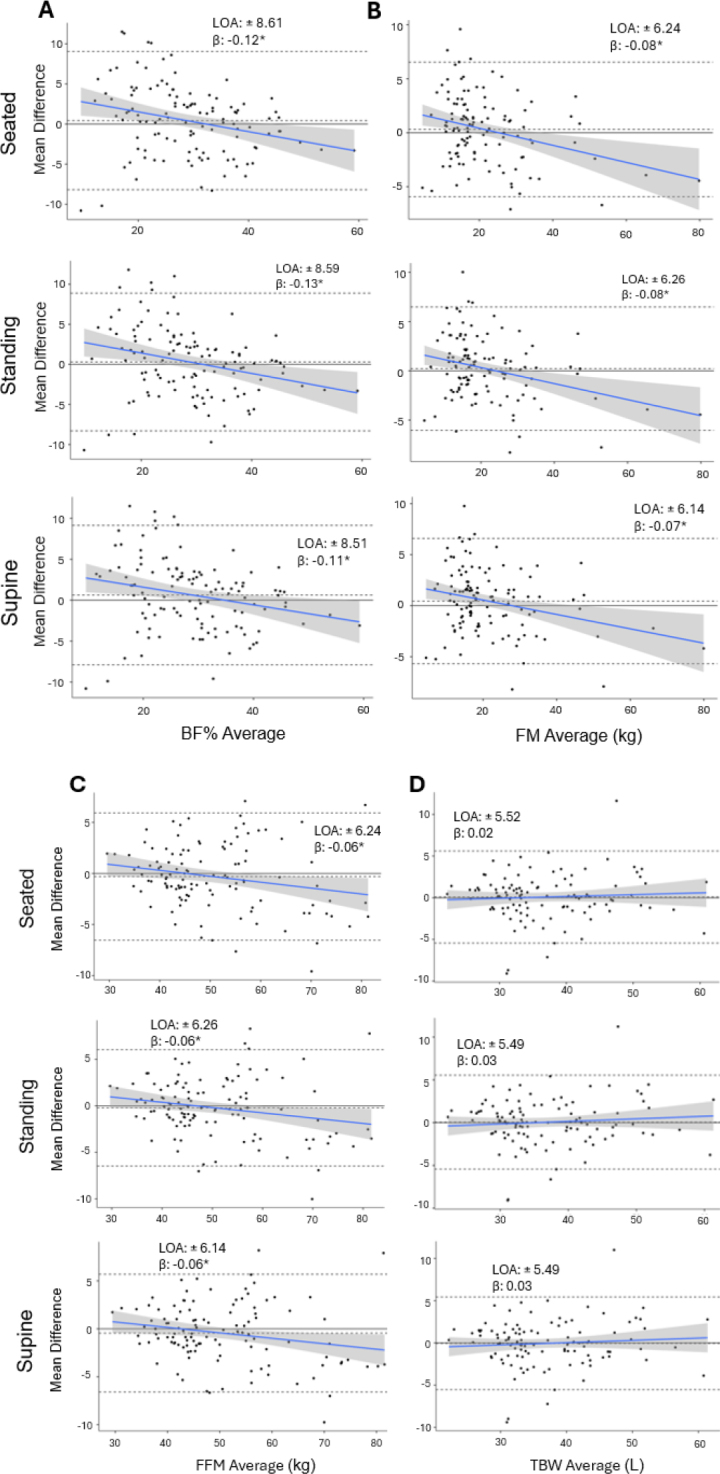
Bland-Altman analyses for body composition and total body water estimates measured by SWBIA and the criterion DXA and BIS in the combined sample. Bland-Altman plots for BF% (a), FM (b), FFM (c), and TBW (d) estimates by SWBIA body position (rows) are displayed. The upper and lower dashed lines on each plot represents the 95% limits of agreement, and the middle-dashed lines represent the mean difference between SWBIA and the criterion DXA or BIS. The solid blue line represents the regression line and the surrounding shading represents the 95% confidence interval of the regression line. SWBIA: smartwatch bioelectrical impedance analysis; DXA: dual-energy X-ray absorptiometry; BIS: bioelectrical impedance spectroscopy; BF%: body fat percentage; FM: fat mass; FFM: fat-free mass; TBW: total body water; LOA: 95% limits of agreement. *, significant proportional bias at p ≤ 0.026.

**Fig 2a-d.**
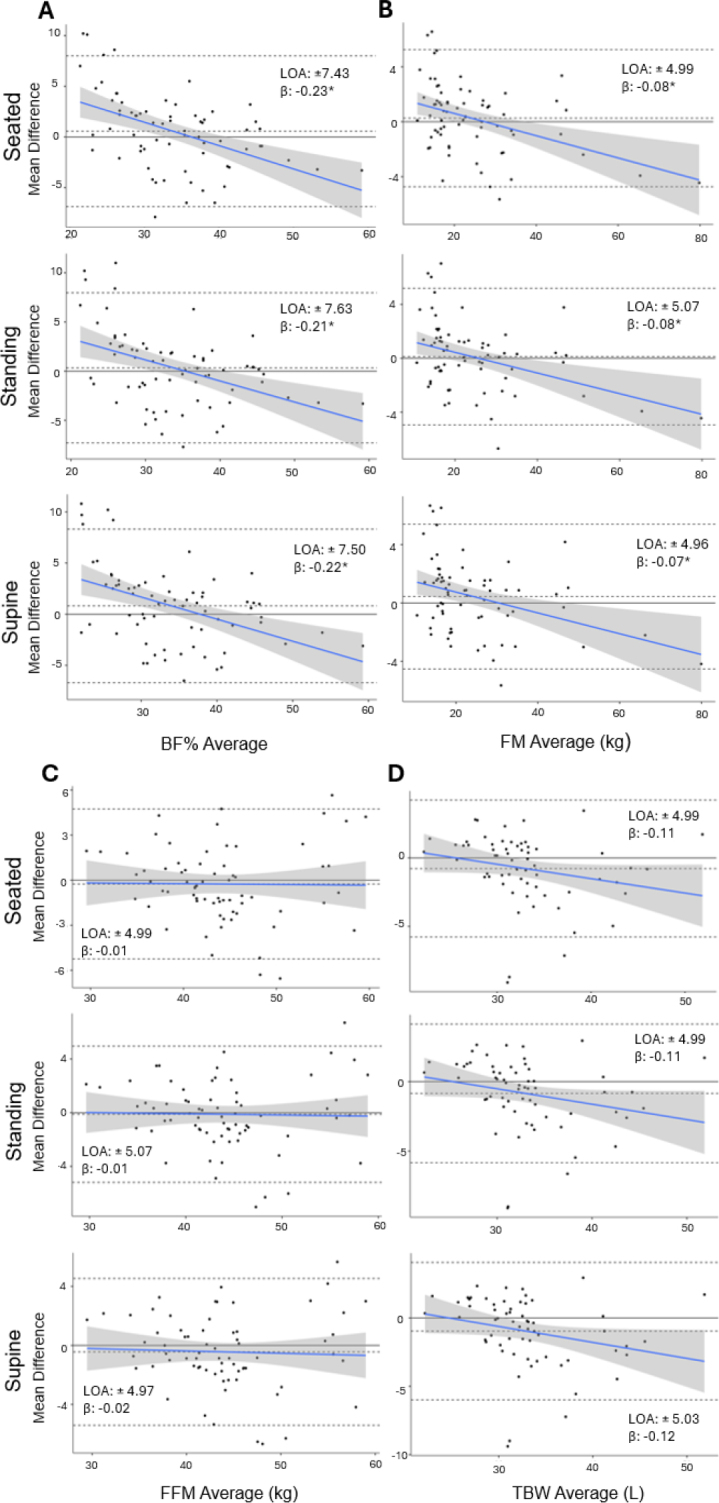
Bland-Altman analyses for body composition and total body water estimates measured by SWBIA and the criterion DXA and BIS in females. Bland-Altman plots for BF% (a), FM (b), FFM (c), and TBW (d) estimates by SWBIA body position (rows) are displayed. The upper and lower dashed lines on each plot represent the 95% limits of agreement, and the middle-dashed lines represent the mean difference between SWBIA and the criterion DXA or BIS. The solid blue line represents the regression line, and the surrounding shading represents the 95% confidence interval of the regression line. SWBIA: smartwatch bioelectrical impedance analysis; DXA: dual-energy X-ray absorptiometry; BIS: bioelectrical impedance spectroscopy; BF%: body fat percentage; FM: fat mass; FFM: fat-free mass; TBW: total body water; LOA: 95% limits of agreement. *, significant proportional bias at p ≤ 0.003.

**Fig 3a-d.**
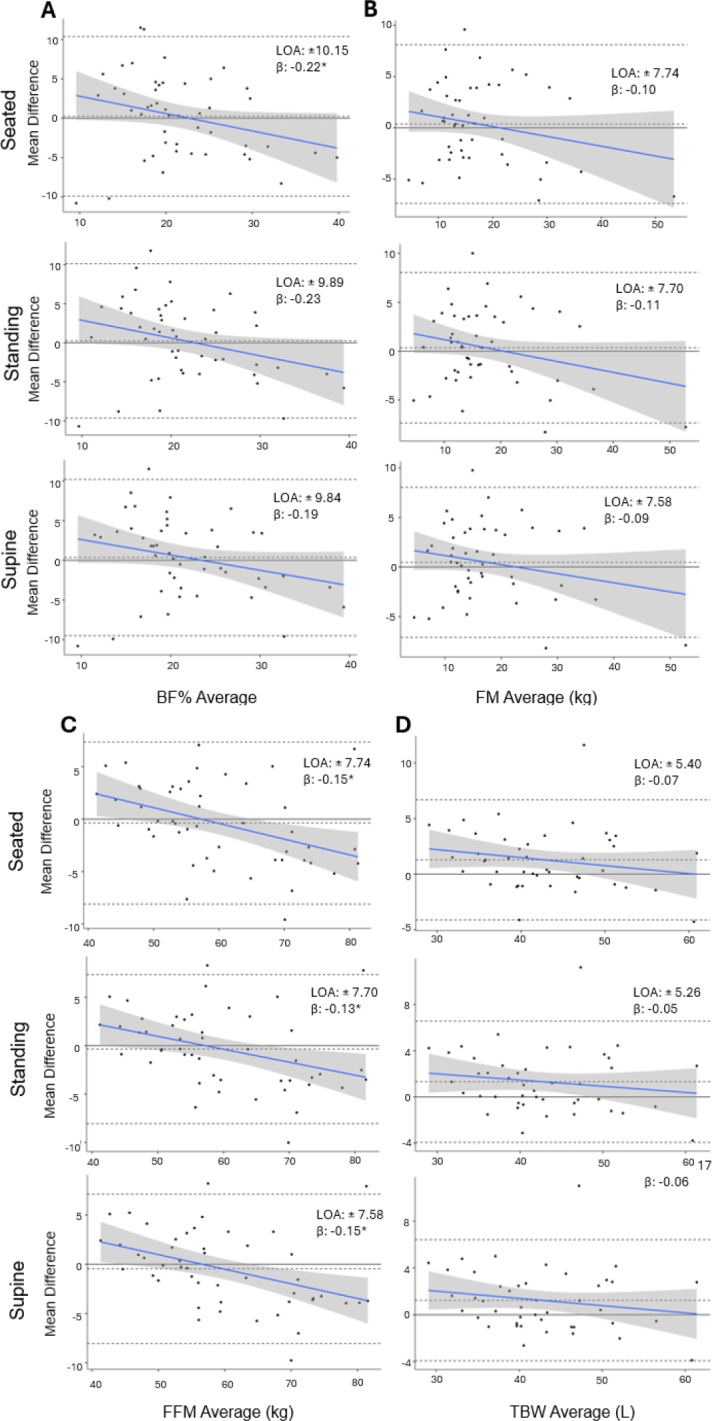
Bland-Altman analyses for body composition and total body water estimates measured by SWBIA and the criterion DXA and BIS in males. Bland-Altman plots for BF% (a), FM (b), FFM (c), and TBW (d) estimates by SWBIA body position (rows) are displayed. The upper and lower dashed lines on each plot represents the 95% limits of agreement, and the middle-dashed lines represent the mean difference between SWBIA and the criterion DXA or BIS. The solid blue line represents the regression line and the surrounding shading represents the 95% confidence interval of the regression line. SWBIA: smartwatch bioelectrical impedance analysis; DXA: dual-energy X-ray absorptiometry; BIS: bioelectrical impedance spectroscopy; BF%: body fat percentage; FM: fat mass; FFM: fat-free mass; TBW: total body water; LOA: 95% limits of agreement. *, significant proportional bias at p ≤ 0.045.

For the combined sample and sex groups, paired null hypothesis significance tests revealed no differences between SWBIA and DXA regardless of body position (**Table 2**; all p ≥ 0.083). For the combined sample and females, both SWBIA FM and FFM demonstrated equivalence with DXA across all body positions; however, only standing BF% demonstrated equivalence with DXA for these groups. No estimate demonstrated equivalence with DXA for males. Results of the Bland-Altman and Deming regression analyses are illustrated in **Figures 1–3** and **Supplemental Figures 1-3**, respectively. In the combined sample, Deming regression revealed no difference from the line of identity for supine FFM estimates only (**Supplemental Figure 1**), whereas differences were observed for all BF% or FM estimates.

RMSEs ranged from 4.37-4.40% for BF% and from 3.15-3.19 kg for FM and FFM; all of which were lowest for the supine position. R2 ranged from 0.82-0.93 and CCC’s ranged from 0.90-0.96; which were similar across body positions. Proportional biases were observed for all estimates in the combined sample (**Figure 1a-d**; all p ≤ 0.026). Large LOA were observed for BF% (±8.51-8.61%) and FM and FFM (±6.14-6.26 kg) with the lowest LOA observed while supine (**Figure 1a-d**).

For females, Deming regression revealed differences from the line of identity for BF% and FM, but not FFM independent of body position (**Supplemental Figure 2**). RMSEs ranged from 3.81-3.89% for BF% and from 2.54-2.57 kg for FM and FFM; all of which were lowest in the seated position. R^2^ ranged from 0.82-0.97 and CCC ranged from 0.88-0.98; all of which were similar between body positions. Proportional biases were observed for BF% (all p < 0.001) and FM (all p ≤ 0.003), but not for FFM for females (all p ≥ 0.741) (**Figure 2a-d**). LOA were large for BF% (±7.43-7.63%) and FM and FFM (±4.96-5.07 kg) for females, with the lowest BF% and FM/FFM LOAs observed while seated and supine, respectively (**Figure 2a-d**).

For males, Deming regression revealed differences from the line of identity for FFM, but not BF% or FM, independent of body position (**Supplemental Figure 3**). RMSEs ranged from 4.98-5.13% for BF% and from 3.85-3.93 kg for FM and FFM, which were lowest in the supine position. R^2^ ranged from 0.56-0.90 and CCC’s ranged from 0.72-0.94, which were similar across body positions. Proportional biases were observed for FFM across all body positions (all p ≤ 0.009) and for standing BF% in males (p = 0.045) (**Figure 3a-d**). LOA were large for BF% (±9.84-10.15%) and FM and FFM (±7.58-7.74 kg) for males with the lowest observed while supine (**Figure 3a-d**).

### Agreement between SWBIA and BIS-derived estimates of total body water by body position

For the combined sample, paired null hypothesis significance tests revealed no significant differences in TBW between SWBIA and BIS (all p ≥ 0.491), and all SWBIA estimates demonstrated equivalence with BIS. However, when separated by sex, SWBIA TBW was significantly higher for males (all p ≤ 0.003) and significantly lower for females (all p ≤ 0.019) across all body positions relative to BIS, and TBW did not demonstrate equivalence with BIS for any position. Deming regression for TBW revealed no differences from the line of identity for all positions in the combined sample (**Supplemental Figure 1**) and while standing and supine and in males (**Supplemental Figure 3**); however, differences from the line of identity were observed across all positions in females (**Supplemental Figure 2**). RMSEs ranged from 2.79-2.80 L for the combined sample, 2.64-2.72 L for females, and 2.88-3.02 L for males; with lowest observed in the seated and supine positions for females and males, respectively.

R^2^ ranged from 0.81-0.89 and CCC’s ranged from 0.88-0.94 across groups and were similar across body positions. No significant proportional biases were observed (all p ≥ 0.050) and LOA were large across all groups and body positions (±4.99-5.52 L); though LOA were lowest for males while supine.

## Discussion

This study sought to determine the differences in SWBIA- derived body composition and TBW estimates, and their agreement with criterion methods, when evaluated across different postural orientations. Given the inherent differences in body composition and fluid distribution patterns between males and females, we also sought to distinguish whether the effect of body position on SWBIA was sex-specific. The major findings from our study were that: 1) body composition and TBW estimates collected in the supine position differed from the estimates collected in standing and seated positions, potentially driven by our female participants; and 2) the agreement between the SWBIA and criterion estimates were generally similar across body positions, though slight improvements were observed when SWBIA assessments were performed while seated and supine for females and males, respectively. Though further evaluation in external and clinical samples are necessary, our findings suggest that SWBIA may demonstrate slight, albeit significant, intra- and inter-device differences in body composition and TBW estimates when measured across postural orientations — and that these differences may vary across sex groups. While sex- and position-specific measurement guidelines could improve the precision of SWBIA, in general, our results highlight the importance of standardized body positioning when using SWBIA. In addition, our findings of similar, yet slightly improved, individual agreement in the seated and supine positions are encouraging for practitioners and clinical populations (i.e., hospitalized patients), as we showed that SWBIA may be employed with confidence in situations where standing may be overly burdensome.

Though our study is the only (to our knowledge) to evaluate the effect of postural orientation on SWBIA using both inferential (intra-device) and agreement (inter-device) analyses, our findings conflict with those from prior investigations reporting no differences in SWBIA precision across body orientations [1]. Specifically, we showed that SWBIA body composition estimates collected in the supine and standing positions were significantly different when evaluated in our combined sample. While this appeared to be driven by our female participants, these intra-device differences were reflected in our inter-device comparisons, where the largest and smallest differences between SWBIA and DXA were observed while in the supine and standing positions, respectively, independent of sex. Given that these positions are diametrically opposed from the clinical perspective, it is unsurprising that we observed these intra-device differences — though studies evaluating more traditional standing and supine BIA modalities have reported in opposition [6].

These intra-device differences, however, do not explain why our inter-device comparisons occurred in accordance. Although no explicit guidelines are provided from the SWBIA interface prior to assessment, previous reports suggest that the developmental models for this technique were generated whilst in seated position [1], and sitting reflects a more *neutral* position relative to both supine and standing. As such, it is reasonable to expect SWBIA to perform best during sitting. Nevertheless, we found that SWBIA estimates produced during sitting revealed no intra-device differences and that the group level agreement with DXA was between the standing (highest) and supine (lowest) assessments.

However, we also showed that supine SWBIA body composition estimates had generally better individual level agreement with DXA. In fact, we found that every body composition variable (other than BF% for females) demonstrated the lowest LOA and RMSE when obtained in the supine position. Further, we found that supine FFM was the only FFM estimate without differences from the line of identity in the combined sample. While it is not uncommon for group and individual level errors to be misaligned, these contradictions may be explained, in part, by the results we observed for TBW. For instance, supine SWBIA TBW estimates in the combined sample were significantly lower compared to sitting and standing estimates; though further analyses revealed that this was driven by seated and supine differences for females. Conversely, no intra-device differences were observed for males, and the supine TBW estimates in males had collectively higher agreement with BIS. Because many BIA techniques use TBW to predict FFM, and because males typically have greater FFM variance, it is possible that the slight improvements in TBW accuracy from the supine position manifested in lower individual error for supine body composition estimates. On the other hand, seated SWBIA TBW estimates performed the worst relative to BIS for males, but the best for females. Like the supine position for males, this may explain why individual error was lower for seated SWBIA body composition estimates for females.

Given the well-demonstrated differences in body composition and fluid distribution patterns between males and females [12,13], it is plausible that hemispheric shifts in body fluid (prompted by our postural adjustments) occurred in a sex-specific manner, and that these shifts led to the intra- and inter-device differences we observed between sex groups. While supine, fluid tends to be evenly distributed across the body, including the regions where females accumulate more FM (lower body) and have lower FFM (upper body). As such, it is possible that supine-related fluid shifts, coupled with the inherent anthropometric characteristics of females, inflated the body composition differences we observed. Prior studies also suggest that standing may underestimate SWBIA body fat estimates in females due to the lower concentration of extracellular fluid in the upper body during testing (i.e. the region most devoid of FFM in females) [3]. Thus, the seated position likely demonstrated the best individual agreement for females because fluid concentrated primarily in the lower body, but remained high enough (in the superior hemisphere of the body) to capture the composition of the upper body. Unlike females in our study, supine SWBIA estimates produced the lowest individual error for males. Because SWBIA injects its currents into the area with the most relative FFM for males (i.e., upper body), the greater distribution of fluid in the lower body while sitting and standing may have resulted in larger FFM underestimation, and greater individual accuracy in the supine position when upper body fluid is most apparent. However, because agreement was generally similar across positions for SWBIA, it is possible that body composition estimates are less sensitive to fluid shifts in males, and that the improved agreement while supine was simply due to the supine orientation of the criterions.

The findings from the present study warrant several considerations when using SWBIA. First, it is important to employ consistent measurement protocols during health monitoring to avoid undue error. To improve clinician confidence and translatability, those using SWBIA assessments remotely should consider adapting their body position protocols to align with those performed in clinical settings. Moreover, given that we showed similar group level agreement across body positions (with slight improvements while seated and supine), our findings demonstrate the potential of SWBIA during periods of immobilization or in situations where standing is overly burdensome. However, given that injury and disease states have their own unique effects on body composition and hydration status, independent of body position, patients and clinicians using SWBIA under these circumstances should exercise caution. Nonetheless, SWBIA may offer patients and practitioners a user-friendly method to monitor body composition and fluid changes with the potential to translate from clinical to remote settings, and future research should consider evaluating the utility of this method in clinical populations.

Our study also had several limitations. Overall, our sample consisted of apparently healthy young adults which limits our ability to generalize our findings in outside groups. We also did not randomize the order of the body positions. However, potential discrepancies due to non-randomization were likely minimized by having participants remain in each position for ≥10 min prior to testing. We also did not remove any excessive body hair from the SWBIA and BIS measurement regions; however, this may be the most practical approach given that most users are unlikely to shave these areas prior testing. Additionally, we did not compare SWBIA TBW estimates to deuterium oxide (the current TBW measurement standard); though TBW estimates from BIS have been validated against those produced using deuterium oxide [8]. Finally, there are no guidelines for arm placement in the supine position and thus, variation in arm orientation while supine may have impacted our findings. Nonetheless, we allowed participants to adjust their arms to a comfortable position to reflect the most likely position outside of a research setting.

In conclusion, our findings suggest that changes in postural orientation may result in slight intra- and inter-device differences in SWBIA body composition and TBW estimates. While sex- and position-specific protocols may lead to modest improvements in accuracy, our findings highlight the importance of standardizing posture when using SWBIA. Given that SWBIA is predominately marketed to the general population as a means of collecting and monitoring health information, it is imperative that standardization procedures, including those for body positioning, are explicitly stated and practiced in order to avoid unnecessary measurement errors.

## Supplemental Files

Supplemental figures: https://github.com/joeb-files/2024_Graybeal
